# Efficacy of a Combination Treatment of Ablative Fractional Carbon Dioxide Laser Therapy and Recombinant Human Epidermal Growth Factor for Atrophic Acne Scars

**DOI:** 10.1111/jocd.16552

**Published:** 2024-08-30

**Authors:** Hao Peng, Xuehui Ran, Xia Yang, Guoyu Zhou, Xiaoxi Lin, Lingyue Shen, Xianglei Wu

**Affiliations:** ^1^ Department of Laser and Aesthetic Medicine, Shanghai Ninth People's Hospital Shanghai Jiao Tong University School of Medicine Shanghai China; ^2^ Department of Oral and Maxillofacial‐Head and Neck Oncology, Shanghai Ninth People's Hospital Shanghai Jiao Tong University School of Medicine, College of Stomatology, Shanghai Jiao Tong University, National Center for Stomatology, National Clinical Research Center for Oral Diseases, Shanghai Key Laboratory of Stomatology, Shanghai Research Institute of Stomatology, Shanghai Center of Head and Neck Oncology Clinical and Translational Science Shanghai China; ^3^ Department of Plastic and Reconstructive Surgery, Shanghai Ninth People's Hospital Shanghai Jiao Tong University School of Medicine Shanghai China

**Keywords:** atrophic acne scar, fractional carbon dioxide laser, recombinant human epidermal growth factor

## Abstract

**Background:**

Atrophic acne scars (AAS) are disfiguring and permanent changes caused by inflammatory acne. Fractional carbon dioxide is a common ablative device used to treat this condition. However, issues such as unclear effectiveness, frequent treatments, and potential side effects exist. In recent years, recombinant human epidermal growth factor (rhEGF) has also been frequently reported for its application in the treatment of acne scars.

**Objective:**

To explore the potential synergistic effect of fractional carbon dioxide laser combined with rhEGF in AAS treatment.

**Methods:**

We enrolled 15 patients with AAS. They received fractional carbon dioxide laser treatment and were then randomly assigned to receive either rhEGF or a placebo on one side of the face. The procedure was repeated three times, and the results were evaluated using the échelle d'évaluation clinique des cicatrices d'acné (ECCA) score and analyzed using the CBS camera system, 3D analysis (3DMD). Reflectance confocal microscopy (RCM) examination was also conducted.

**Results:**

Both sides exhibited significant improvement in the appearance of the acne scars after treatment, as confirmed by the ECCA score, 3DMD data, and CBS texture score. On the rhEGF‐treated side, the pore number and epidermal pigment area significantly improved as compared to the control side, whereas no significant differences were observed in the other data. Under RCM, a significant increase in epidermal thickness and appearance of reticular collagen fibers in the dermal layer after treatment was observed.

**Conclusion:**

Compared to the sole use of laser, the combination of fractional carbon dioxide laser and rhEGF does not significantly enhance scar therapeutic effects. However, it does shorten the recovery period after laser treatment and improves the pore appearance.

## 1 Introduction

An atrophic acne scar (AAS) represents a prevalent disfiguring condition characterized by dermal tissue damage primarily induced by severe acne [1]. Currently, the foremost frontline therapeutic modalities employed in clinical practice encompass chemical peeling, laser therapy, dermal fillers, dermabrasion and tissue augmenting agents [2]. As an ablative laser, fractional carbon dioxide laser can effectively generate microscopic thermal zones in the epidermis and dermis of affected skin, triggering wound healing and facilitating skin remodeling [3]. However, at times, a singular therapeutic approach may yield suboptimal outcomes for deep scars like V‐shaped icepick scars [4]. Thus, enhancing the treatment efficacy, such as combination therapy and adjuvant medication, remains a pressing issue requiring resolution. Additionally, it may result in skin sensitivity postoperatively. For individuals with dark skin, there might be prolonged issues related to post‐inflammatory hyperpigmentation [5]. Consequently, mitigating postoperative complications is also a matter worthy of consideration.

Epidermal growth factor (EGF), a protein composed of platelets, macrophages, and monocytes, controls the growth and differentiation of keratinocytes as well as dermal fibroblasts [6]. Recently, it has been reported that the early use of EGF could improve cutaneous wound healing [7]. Recombinant human epidermal growth factor (rhEGF) greatly reduced skin scarring by inhibiting the inflammatory response, reducing the transforming growth factor (TGF‐β1) expression, and inhibiting excessive collagen formation during healing of full‐thickness wounds in mice [8]. Particularly, EGF alone reportedly can improve acne and acne scars [1, 9, 10]. Previously the auxiliary use of rhEGF in treating stretch marks after carbon dioxide fractional laser surgery showed a considerable improvement [11], suggesting that acne scarring could be improved better with treatment combination of rhEGF and fractional CO_2_ laser. However, a previous study showed that, among healthy individuals who used EGF after ablative carbon dioxide laser therapy, the transepidermal water loss and PIH on the experimental side were not significantly difference with those on the control side [12]. The conclusions need to be further confirmed.

The present study aimed to investigate whether the adjunctive use of rhEGF after fractional CO_2_ laser treatment of AAS could enhance the efficacy of phototherapy and reduce the incidence of postoperative adverse effects.

## 2 Materials and Methods

In this double‐blind, split‐face clinical trial, 15 patients with AAS who were admitted between July 2022 and November 2023 were enrolled. They had never received treatment before recruitment. The exclusion criteria were those with severe active acne, keloids, and hypertrophic scars. This study was approved by the ethics committee. A written informed consent for study participation was obtained from each patient.

Patients received three fractional CO_2_ laser treatments at 3‐month intervals. The device used was Ultrapulse (Lumenis, Santa Clara, CA) with the following parameters: 20–40 mj and 10%–15% coverage using DeepFx mode. The patients were anesthetized with a mixture of 2.5% prilocaine and 2.5% lidocaine cream (Ziguang Co. Ltd, Beijing, China) pretreatment. Cold compresses were used to the treated area immediately after surgery until pain and discomfort subsided. Patients were told to avoid washing their face for 7 days. Moreover, for the next month, patients were asked to use an rhEGF spray, which was prepared using a 40 000‐ng rhEGF dissolved in 15 mL of purified water, on one side of the face and a saline spray on the other side one spray every time, three times a day for 1 month.

Patients were evaluated before each treatment and 3 months after final treatment. Standardized photographs were taken at every visit using CBS‐Dermoscopy (CBS, Taiwan). Clinical efficacy was assessed using the échelle d'évaluation clinique des cicatrices d'acné (ECCA) score by two independent dermatologists. The AAS changes were also measured by evaluating three‐dimensional images obtained using the 3DMD imaging system (Atlanta, GA, USA), which represents loss of skin tissue. All patients noninvasive RCM (Vivascope 3000; Lucid Inc., Rochester, NY) examination at every visit. Vivastack mode images were collected at three sites for each side of the face. The epidermal thickness was measured using this mode, extending from the top of the skin to the top of the dermal papillae. Meanwhile, the morphology and changes of the collagen fibers in the dermis were also be recorded. Furthermore, each patient was asked to complete the Sensitive Scale‐10 (SS‐10) questionnaire at 1 week after treatment and to record the number of days of scab shedding on each side of face.

All statistical analyses were conducted using a designated software (SPSS 25.0; IBM Corp., Armonk, NY) and GraphPad Prism 9 (GraphPad Software Inc., San Diego, CA). The Wilcoxon signed‐rank test was used to compare the treatment results. Statistical significance was set at *p* < 0.05.

## 3 Results

**FIGURE 1:**
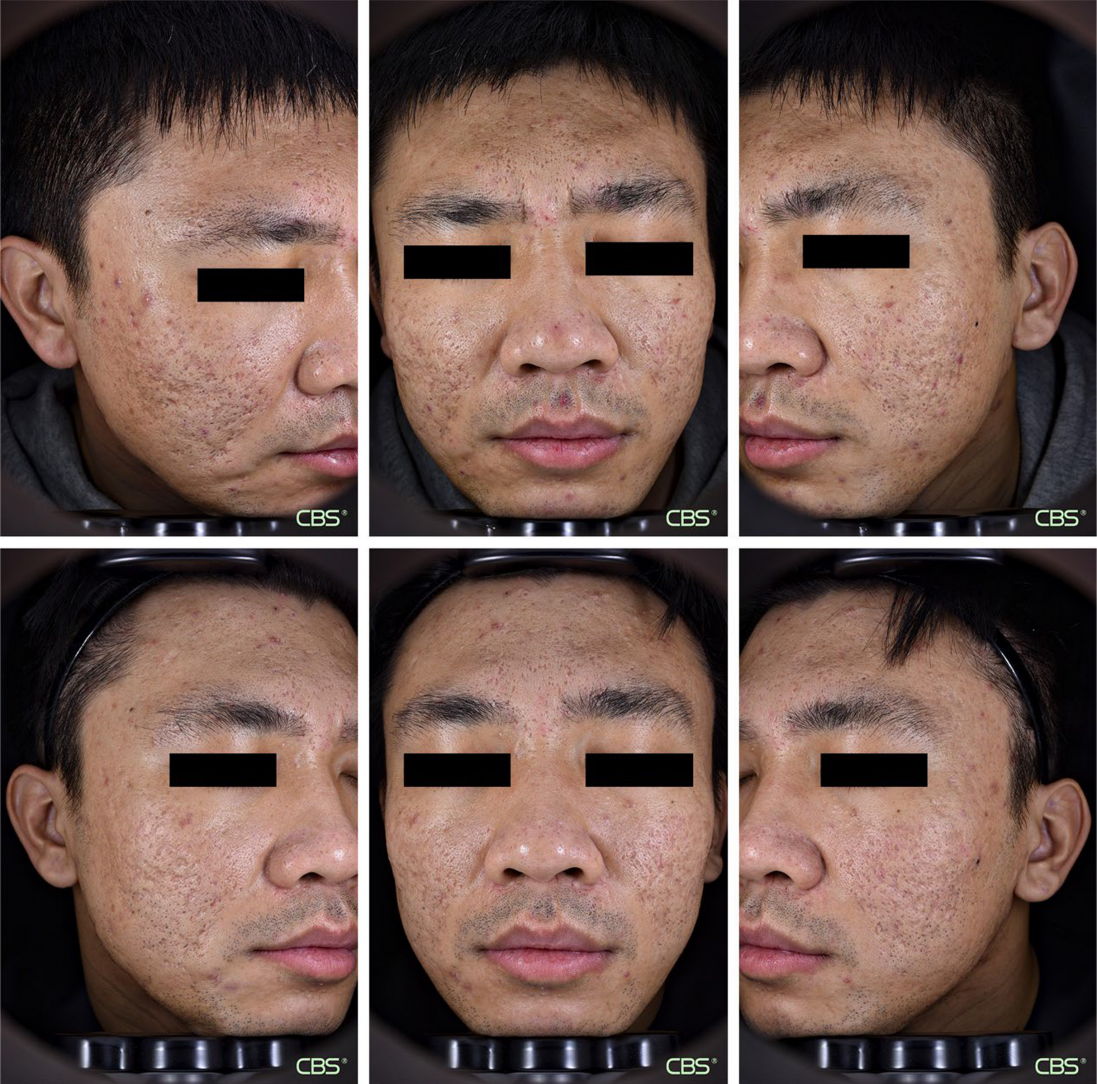
Clinical photographs of AAS patients taken at baseline (upper row) and at 3 months after the third treatment (lower row) show clinical improvements on both the treatment (R) and control (L) sides. AAS, atrophic acne scar.

Altogether, 15 patients aged 26–46 years (34.56 ± 6.23 years; 8 men and 2 women) with Fitzpatrick skin types III–IV were enrolled in the present study. The ECCA scores were 100.00 ± 38.73 and 92.22 ± 36.41 on the rhEGF and control sides, respectively, at baseline, showing no significant difference. After three treatment sessions, the AAS on both sides improved; the scores on the rhEGF and control sides were 71.11 ± 27.81 and 67.78 ± 26.35, respectively, showing statistical significance (*p* < 0.05). The extent of improvement observed before and after treatment on the rhEGF side was not significantly superior to that of the control side (Figure 1).

**FIGURE 2:**
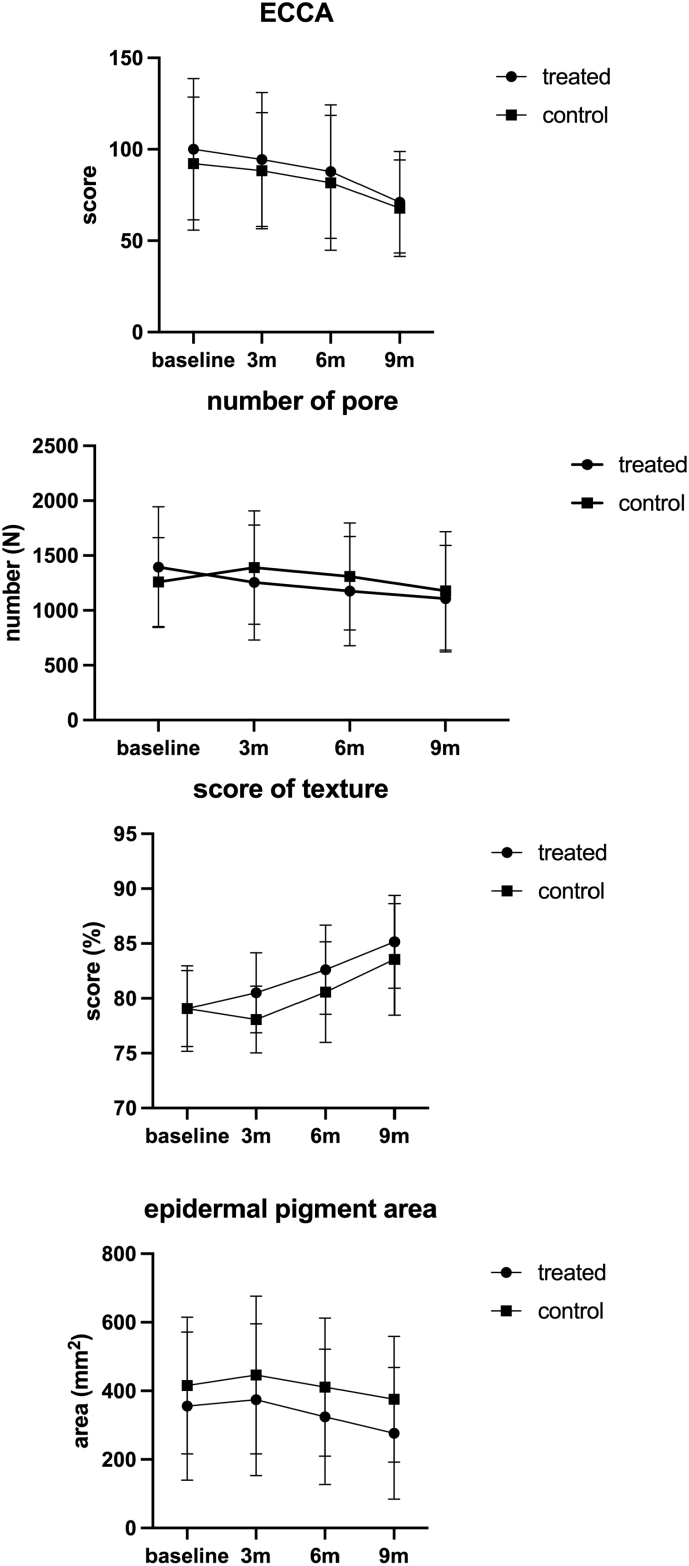
ECCA scores, number of pores, texture score, and epidermal pigment area calculated by using the CBS system at baseline and at 3 months after the third treatment for the rhEGF‐treated and control sides. ECCA, échelle d'évaluation clinique des cicatrices d'acné; rhEGF, recombinant human epidermal growth factor.

**FIGURE 3:**
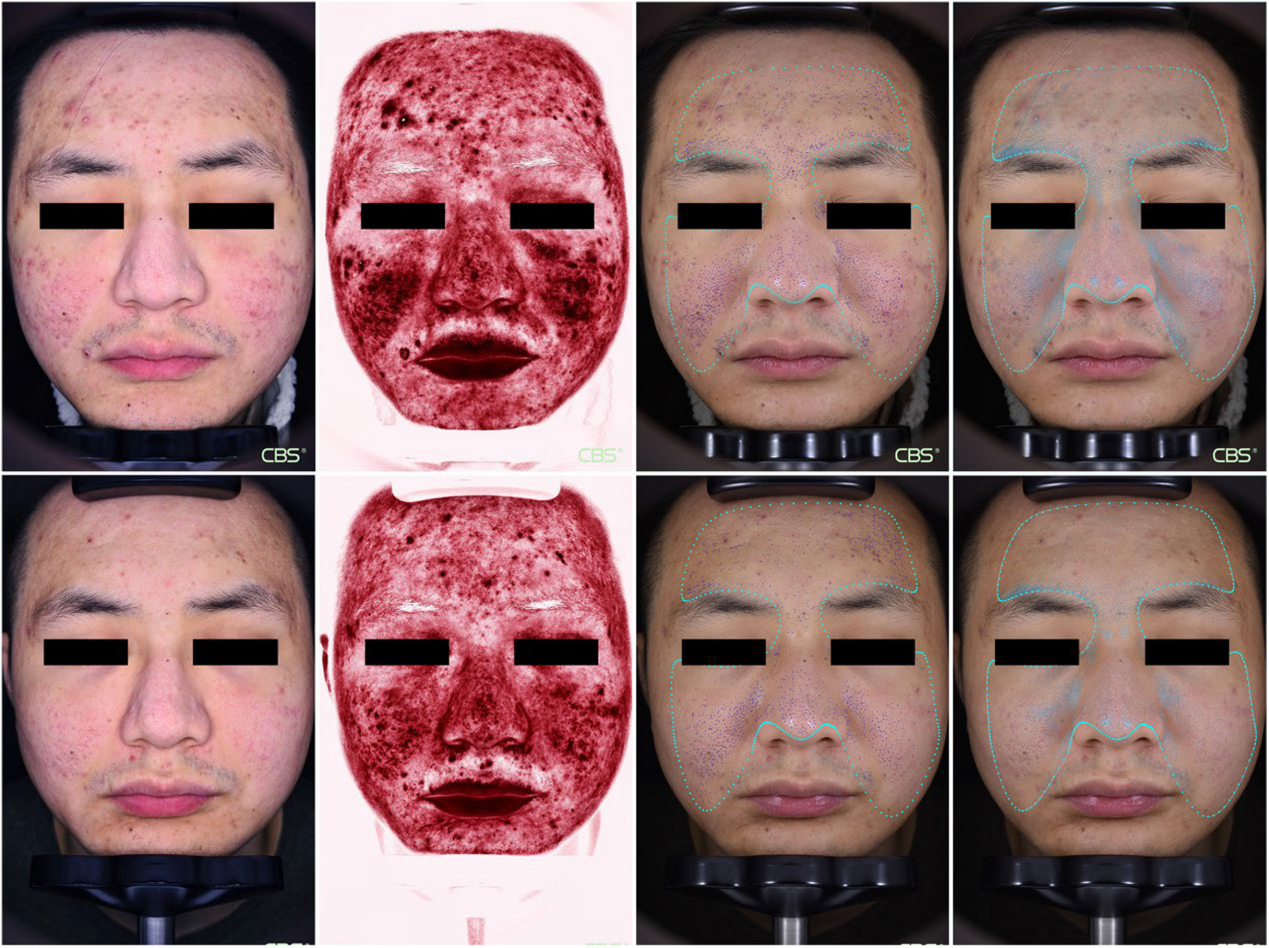
CBS images of a 27‐year‐old male taken preoperatively (upper row) and 3 months after the final treatment session (lower row) are presented. The patient's left side received rhEGF treatment, while the right side served as the control. The images were captured using polarization mode and red area analysis, and pore and smoothness analyses were conducted. The results demonstrate an improvement in the patient's AAS, characterized by a reduction in the red area, a decrease in the number of pores, and an enhancement in smoothness. AAS, atrophic acne scar; rhEGF, recombinant human epidermal growth factor.

The CBS camera system evaluated the patients before and after treatment. The texture score of both the rhEGF and control sides was significantly improved after three treatments, with no difference between the two sides. Regarding the pores, the number of pores decreased only on the rhEGF side, whereas no significant change was seen on the control side. Similarly, the epidermal pigment area of the rhEGF side decreased from 355.98 ± 216.30 to 276.36 ± 191.93 after treatment. The changes between the two sides were statistically significant (Figures 2 and 3).

**FIGURE 4:**
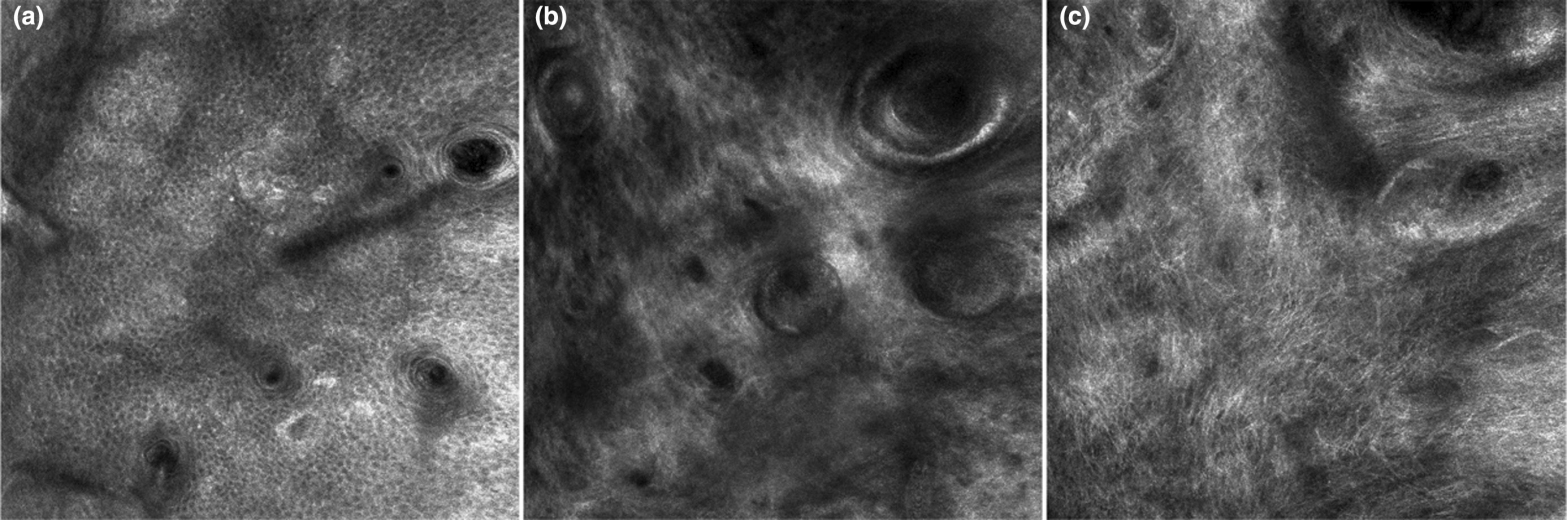
RCM images reveal a honeycomb‐like structure in the epidermal layer, with a small amount of highly refractive melanin (a), the dermal layer contained coarse collagen fibers before treatment (b), whereas after treatment, a reticular arrangement of fibers became visible (c). RCM, reflectance confocal microscopy.

Through reflectance confocal microscopy (RCM) examination, we observed a significant increase in the epidermal thickness on both sides of the face after treatment. On the rhEGF side, the thickness increased from 30.24 ± 2.22 to 36.47 ± 6.98 μm, but no significant difference in epidermal thickness was observed between the two sides. RCM examination showed that coarse collagen could be seen at the dermis before treatment and was replaced by reticulated collagen fibers arranged in a net after treatment (Figure 4).

**FIGURE 5:**
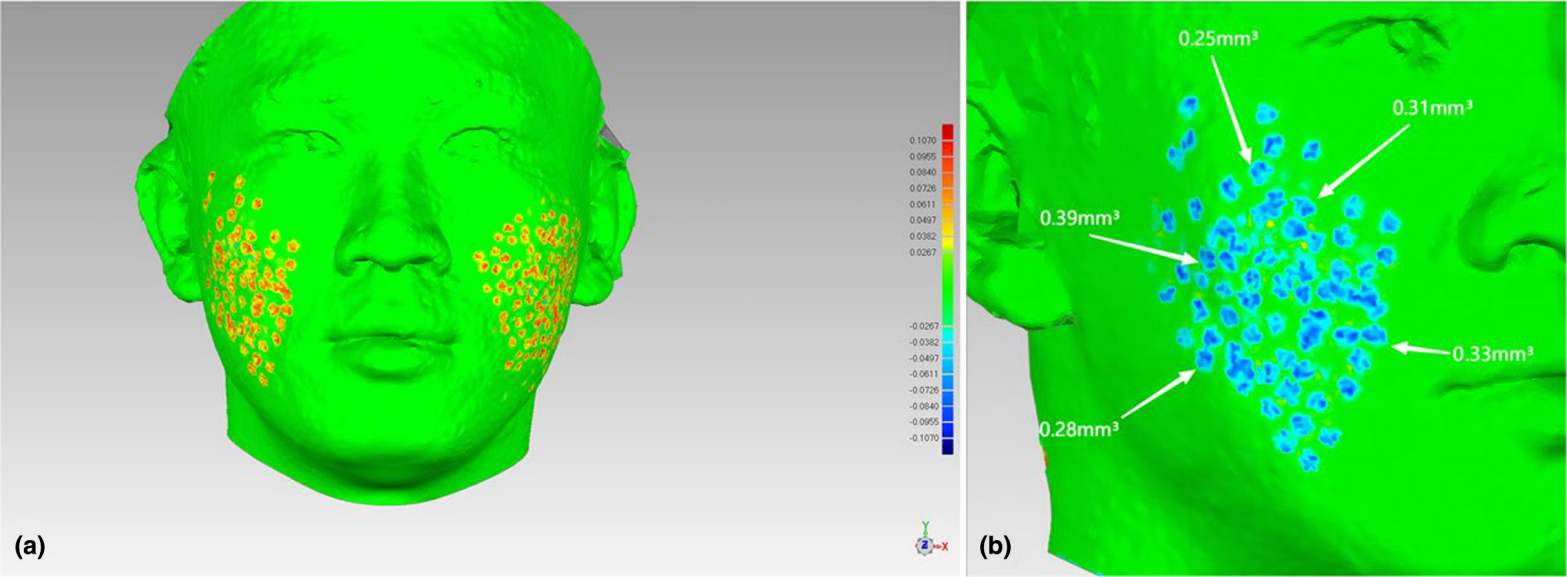
Following the analysis of 3DMD data, (a) illustrates the preoperative data of localized AAS, (b) presents a comparative chart depicting the difference between 3 months postoperatively and preoperatively. AAS, atrophic acne scar.

Through the analysis of 3DMD photographs taken before and after treatment, both the rhEGF and control sides showed statistically significant improvements in AAS. The improvement on the rhEGF side was 12.96 ± 3.84 mm^3^, whereas that on the control side was 12.11 ± 5.03 mm^3^, showing no significant difference between the two groups (Figure 5).

The durations of scab shedding were 4.33 ± 0.71 and 5.85 ± 0.70 days on the rhEGF and control sides, which significantly reduced the recovery time. No statistical difference was found in the SS‐10 scale scores on the seventh day after the treatment between the two sides.

## 4 Discussion

Atrophic acne scars are relatively common in adolescents and adults with acne, which may cause aesthetic and psychological problems [13]. It is caused by the excessive release of matrix metalloproteinases in the dermal layer, leading to the degradation of collagen fibers [14]. Fractional CO2 laser, by stimulating the regeneration of collagen in the dermal layer, proves to be an effective therapeutic approach [15]. However, problems still exist, including long recovery period, multiple treatments, and unclear effect [16].

Epidermal growth factor has a clinical impact on wound healing by stimulating keratinocyte proliferation and acting on fibroblasts and smooth muscle cells to shorten the healing time and increase the tensile strength [13]. In rat wound models, Qi et al. [17] found that EGF was released more in oral mucosal wounds than in skin wounds, resulting in faster healing and less scarring in the former. EGF may be involved in scar formation. Ryu et al. [18] manually confirmed that the topical application of rhEGF ointment after surgery can shorten the length of the surgical wound and reduce the wound are as compared to antibiotic ointment, but no difference in the melanin and erythema indices were observed between the treatment sides. However, in our experiment, that the epidermal pigmentation exhibited a noticeable reduction on the treated side as compared to the control side, following three sessions of fractional carbon dioxide laser therapy. This finding aligns with the results obtained by Ratanapokasatit [19]. It may be attributed to the potential role of EGF in decreasing inflammation‐induced melanin production [20]. We also attempted to quantitatively analyze the amount of epidermal pigment granules in the RCM images using Otsu's method [21] by calculating the most refractile substance in the spinous layer image as melanin. However, we encountered difficulties in achieving uniform image brightness, requiring the development of a better method for calculation.

Recently, some reports have highlighted the use of topical application of EGF for improving AAS. After 4 weeks of EGF cream application, a significant improvement in inflammatory acne lesions and a reduction in the occurrence of AAS have been observed in a previous study [22]. Kim further discovered that the use of EGF can stimulate the release of TGF‐β1, elastin, and Type 1 and 3 collagen. Storddard [10] have also confirmed that topical EGF application can improve the appearance of scars caused by inflammatory acne. Moreover, in Seidel's experiment [23], the use of EGF for 12 weeks also revealed remarkable improvements in AAS. However, in our own experiment, similar changes were not observed. Compared to the side treated solely with fractional CO_2_ laser therapy, the use of rhEGF did not result in significant changes in the ECCA or objective texture score as well as 3D statistics for AAS. However, a notable advantage was observed in the objective pore score when rhEGF was used in combination with fractional CO_2_ laser treatment. This could be attributed to the stimulation of fibroblasts by rhEGF, resulting in the production of a significant amount of collagen. As a result, this increase in collagen might contribute to the thickening of the dermis, thereby improving the appearance of certain aging‐related pores [24]. However, the amount of collagen produced is still insufficient to further improve atrophic scars.

However, our study still has some limitations. As a single‐center study with a relatively small sample size, the final follow‐up was conducted at 3 months. Moreover, there was a lack of fundamental research or microscopic evidence.

## 5 Conclusions

Our study findings revealed that the application of fractional CO_2_ laser treatment can significantly improve the appearance of AAS. After three treatment sessions, there was a notable reduction in the number of pores on the rhEGF side, as compared to the control side. The enhancing effect on depressed scars, however, was not as pronounced. Additionally, on the experimental side, there was a significant acceleration in scab shedding posttreatment, and the risk of post‐inflammatory pigmentation decreased. Therefore, combining rhEGF with fractional CO_2_ laser treatment post‐procedure can expedite the recovery, reduce the occurrence of adverse reactions, and provide auxiliary benefits in improving pore appearance.

## Author Contributions

H.P., X.W., X.Y., and X.R. performed the research. H.P. and X.W. analyzed the data. L.S. and G.Z. assessed the efficiency. L.S., W.X., and X.L. conceived and designed the study. H.P., X.W., and L.S. wrote the paper.

## Ethics Statement

This retrospective study was approved by the ethics committee of Shanghai Ninth Hospital.

## Conflicts of Interest

The authors declare no conflicts of interest.

## Data Availability

The data that support the findings of this study are available from the corresponding author upon reasonable request.
